# Editorial for Special Issue: “MicroRNA in Cardiac Health and Disease”

**DOI:** 10.3390/ijms232415567

**Published:** 2022-12-08

**Authors:** Francesca Forini, Letizia Pitto

**Affiliations:** CNR Institute of Clinical Physiology, Via G. Moruzzi 1, 56124 Pisa, Italy

MicroRNAs (miRNAs) are endogenous, evolutionarily conserved, non-coding RNA molecules that influence most, if not all biological events, with cardiovascular development and homeostasis being no exceptions. Since their discovery just over 20 years ago, a number of critical cardiac-specific miRNAs have been identified, and several miRNAs have been shown to be expressed in a dysregulated manner during heart disease progression. We now know that miRNAs are post-transcriptional regulators of gene expression in tightly regulated cardiac development and in major cardiac physiological and pathological processes, such as contractility, arrhythmia, myocardial infarction, hypertrophy, and inherited cardiomyopathies.

This Special Issue explores three main aspects of miRNA function in cardiac pathophysiology: (1) their role as predictive and diagnostic biomarkers, (2) the potential of miRNA-based therapeutic and reparative strategies, and (3) the unraveling of novel miRNA/gene regulatory circuits involved in disease progression.

Manetti et al. [1] discuss the involvement of miRNAs in sepsis-induced cardiac disease. Their systematic review of the relevant literature unveils altered levels of 77 miRNAs assessed in both in vivo and in vitro experimental models or in the peripheral blood of septic patients with cardiac impairment. The most relevant transcripts are involved in critical processes, including immunomodulation (miR-187, -155, -214, -21, -31), inflammation (miR-27, 125a, -130, -210, -127, -150, -342, -146a, -146b), endothelial activation (miR-181b, -146), mitochondrial damage (miR-202-3p), and cardiac dysfunction (miR-20b-3p, -155-3p, -155-5p, -25, -223, 146b). The vast majority of dysregulated miRNAs converges upon the activation of nuclear factor-κB (NF-κB), an inducible transcription factor that regulates the pivotal mediators of the innate and adaptive immune response and inflammation. As an interesting finding, a lot of the reported miRNAs are also involved in atherosclerosis, which is consistent with the contribution of infections and inflammation in plaque development. In the tangled forest of interconnected signaling pathways, miR-223 and -23b are regarded by the authors as the most relevant due to their broader implication in other pathologies leading to myocardial impairments such as coronary artery disease (CAD) and cardiac fibrosis. Focusing on CAD, the narrative review of Kong et al. [2] addresses the potential role of circulating miRNAs as biomarkers of ischemic heart disease (IHD). When compared to other commonly used non-imaging proteins, such as cardiac troponins and c reactive protein, miRNAs exhibit a higher sensitivity and greater power to detect early stages of the disease. The main advantage of circulating miRNAs is that they are involved in every step of the atherosclerotic process namely: endothelial cell dysfunction and monocyte infiltration (miR-370, -145, -182-5p, -9-5p, -451b, -381, -146a, -126), the activation of monocytes and differentiation of macrophages (miR-155, -23a-5p, -320b), plaque angiogenesis (miR-342-5p, -21), vascular smooth muscle cell proliferation and differentiation (miR-30b-5p, 574-5p), fibrous cap destabilization and plaque rupture (miR-21, 123-3p). Despite promising findings, the use of miRNAs as diagnostic and prognostic tools in cardiac patients still requires further research and standardized protocols. To guide the identification of good candidates to test in the clinical practice, a criterion clearly emerged from the Special Issue is the greater predictive power of miRNAs that are dysregulated in entire disease processes or that are shared between different disease triggers rather than miRNAs only involved in one particular aspect of the disease evolution [3,4,5]. For example, Jayawardena et al. [3], performed an independent analysis of the Human microRNA Disease Databases (HMDD) to retrieve common dysregulated miRNAs associated with ischemia and reperfusion (IR) injury and other cardiac disease in humans and animal models. This in silico analysis, in conjunction with a literature search, led to the identification of miR-21 and miR-1 as shared terms in IR, myocardial infarction and CAD, which can be further explored in a future clinical translation. Along the same priority principle, in the context of human atrial remodeling, the original article by Djalinac et al. [4] used ex vivo human models of atrial stretch and tachycardia, combined with microarray gene expression profiling, to identify common transcripts induced by the two different atrial disease triggers. Though stretch and tachycardia elicit distinct transcriptomic signatures, the authors identify the miR-1183 precursor as the common highest upregulated molecule. The finding is also confirmed in chronic conditions in atrial and ventricle samples from patients suffering from long-term atrial fibrillation or end-stage dilated cardiomyopathy, suggesting either a key role of the miRNA as a tissue biomarker, and its potential functional significance in cardiac disease. Notably, the overexpression of miR-1183 upon stretch and tachycardia corresponds to a downregulation of two predicted miRNA targets: ADAM Metallopeptidase Domain 20 (ADAM20) and Lipoprotein-Associated Phospholipase A2 (PLA2G7). While the role of ADAM20 in cardiovascular disease remains to be explored, PLA2G7 is shown to mediate detrimental proinflammatory, atherosclerotic, and profibrotic processes in myocardial remodeling [6,7,8]. On these bases, the authors conclude that miR-1183 upregulation may represent a naturally occurring protective mechanism against cardiac remodeling, fibrosis, and aging. Accordingly, previous studies demonstrated the compensatory and adaptative role of miRNA overexpression against noxious processes, such as the case of miR-29 in aging-associated neural disorders and cardiac disease [9,10].

An interesting part of the Special Issue is dedicated to the potential use of miRNAs for the development of oligonucleotide-based therapeutic strategies [2,3,5]. The paper by Alam et al. [5] focuses on recent advancements and emerging perspectives for cardiac repair in post-ischemic cardiac disease and heart failure (HF). In this narrative, miRNAs are described as the critical mediators of the protective effects of either cell-based and cell-free strategies. Several cell types with different origins and functional characteristics have been tested in preclinical models, with encouraging results related to infarct size reduction and functional recovery. When advanced to clinical trials, these approaches demonstrated modest but significant benefits against major adverse cardiac events and improved quality of life [5]. The general consensus is that the favorable outcomes observed upon cell transplantation depend on indirect, paracrine signals favoring neoangiogenesis and cardiomyocytes survival. It is now clear that these effects are largely promoted by miRNAs transported in great quantity in stem-cell-derived exosomes. As a consequence, the exosome-mediated delivery of protective miRNAs has recently been appraised as a better translatable option for cell-free approaches to cardiac repair [5]. Even though miRNAs are promising targets for personalized medicine and improved cardiac patient care, manipulating their expression for therapeutic purposes requires overcoming several challenges that are highlighted in the Special Issue [3,4,5]. Major difficulties are the specific delivery of the clinically relevant amount of miRNAs at the site of myocardial injury, avoiding off-target effects, causing unwanted toxicity and adverse outcomes, and preventing immunological activation [3,4,5].

Finally, the original article by Canale et al. reveals a new regulatory axis whereby the silencing of deiodinase-3 (Dio3), under the control of the myomiR-133a-3p, drives the thyroid hormone (T3)-dependent activation of the protective mitoK-ATP channel in the post-IR setting [11]. Consistent with previously published research, in the preclinical model, IR injury induces a significant reduction in miR-133a associated with an overexpression of Dio3, the thyroid hormone inactivating enzyme. While this protein plays a crucial role during fetal life to promote cell proliferation and protect the developing heart from thyrotoxicosis-derived abnormalities [12,13], its reactivation under disease conditions leads to a detrimental reduction in T3 levels, known as a low T3 state (LT3S), which favors a maladaptive remodeling of the heart in the long term. An early correction of the LT3S, via T3 replacement at physiological concentrations, produces a normalization of the cardiac levels of miR-133a, thus re-establishing the inhibition of Dio3 expression, and allowing the transcriptional reactivation of the T3-sensitive genes mitoK and mitoSur, the recently identified constituents of the mitok-ATP channel. Aside from adding novel information on the mitochondria-targeted cardioprotective effects of T3, the identification of a T3/mir-133/Dio3 axis could inspire future regenerative strategies. Indeed, adult cardiomyocytes are almost terminally differentiated cells and the experimental attempts force their proliferation with miRNA-based approaches, resulted in mitotic catastrophe [5]. Given the critical contributions of T3, miR-133a and Dio3 in the processes of cell proliferation/differentiation/transdifferentiation, a better understanding of their interaction and underlying mechanisms might allow their levels to be manipulated in the injured myocardium to achieve the efficient proliferation of adult cardiomyocytes, either in cell-based and cell-free strategies.
